# Decolonising medical causality in the COVID-19 pandemic

**DOI:** 10.4102/sajid.v37i1.347

**Published:** 2022-02-28

**Authors:** Adele Visser

**Affiliations:** 1 Department of Philosophy, Practical and Systematic Theology, School of Humanities, University of South Africa, Pretoria, South Africa

**Keywords:** COVID-19, causality, communitarianism, African philosophy, decolonisation

## Abstract

The coronavirus disease 2019 (COVID-19) pandemic has irrevocably changed every aspect of social, medical and economic life globally. Although our traditional Western consideration of the underlying causes have led to massive strides in prevention and control of spread, a wider more inclusive approach, including principles of African and non-Western causality may facilitate our ability to prevent future outbreaks. Decolonising our traditional thoughts on medical causality may compliment the practice of medicine and enrich our understanding of health.

The global pandemic caused by severe acute respiratory syndrome coronavirus 2 (SARS-CoV-2) is likely the most significant challenge to face our generation. Understanding this crisis may counter-intuitively be more productive if we apply the Eisenhower principle, whereby a problem is approached not by breaking it into small parts, but rather to make it bigger.^1^ Modern medical pathogenesis is heavily influenced by Humean principles where the constant conjunction between putative cause and putative effect, together with necessary connection, uniformity and universality suggests causality.^2,3^ Using this paradigm, we can simply state that the coronavirus disease 2019 (COVID-19) pandemic is caused by the spread of a respiratory virus, leading to an initial viral response, followed by an inflammatory second phase.^4^ Therefore, in order to regain control over this outbreak, we need restricted spread through social distancing, mask wearing, prevention by vaccination and cure by effective treatment.

Troxell and Snyder^5^ published a case study in causality using the example of a house fire caused by a youth who played with matches. According to fire fighters, the cause of the fire was the matches. However, a physicist may state that this was not sufficient and the presence of ample combustible material was the cause. A psychologist may query this analysis by stating the mental well-being of the child as cause whereas an anti-smoking activist may lay blame on the free availability of matches to those too young for judicious use. Of course, this analysis can go on ad infinitum. The important point is to show that none of these causalities are right or wrong, or even more or less right, but rather that they are complementary.

Using principles of African philosophy may facilitate the process of true root-cause analysis of our current pandemic. African metaphysics do not subscribe to the dualist nature of Western models. There is no distinction between God, the ancestors, people and nature and all these parts of a universal whole is used in the ontological explanation of events such as illness or disaster. There is acknowledgement of certain aspects within this universal complexity to be beyond our perceptual capabilities.^6^ In African culture, illness is also more broadly defined as a state of not being able to function normally, not simply the absence of health. It therefore considers not only physical, psychological and social factors but even environmental and moral cause.^7^ The ethic of care and ethic of nature-relatedness emphasises the importance of responsibility of humans in the upkeep of the environment.^8^

If we critically consider these principles in the assessment of causality in the COVID-19 pandemic, the importance of underlying chronic diseases and comorbidities carry far more weight and social support and communitarianism. In addition, the infringement in harmony between humans and their environment becomes central in the management of the crisis. The unnatural and stressful environment of wet markets, contravene fundamental principles of African harmony.

Both Western and African causality should be used in complementary fashion in our evaluation of the current crisis facing the world, to enable a comprehensive and lasting attempt to prevention.
